# Unsupervised Assessment of Frailty Status Using Wearable Sensors: A Feasibility Study among Community-Dwelling Older Adults

**DOI:** 10.1177/27536351241311845

**Published:** 2025-02-15

**Authors:** Oonagh Mary Giggins, Grainne Vavasour, Julie Doyle

**Affiliations:** NetwellCASALA, Dundalk Institute of Technology, Dundalk, Co Louth, Ireland

**Keywords:** Frailty, older adults, wearable sensor, remote monitoring

## Abstract

**Objectives:**

This study examined whether community-dwelling older adults can independently capture wearable sensor data that can be used to classify frailty status.

**Methods:**

Fifty-one older adults (age 77.5 ± 8.4 years, height 163.6 77.5 ± 8.4, weight 72.0 ± 13.5 kg, female 76%) took part in this investigation. Participants independently captured physical activity and physical function data at home using a smartwatch and a research-grade inertial sensor system for 48-hours. Machine learning classifiers were used to determine whether the data obtained can discriminate between frailty levels.

**Results:**

Models incorporating variables from both the smartwatch and inertial sensor system were successful in the prediction of frailty status.

**Discussion:**

This study has demonstrated the ability of older adults to collect data which can be used to indicate their frailty risk. This may enable earlier intervention and lessen the impact of frailty on the individual and society as a whole.

## Introduction

The world’s population is ageing at an unprecedented rate. According to estimates from the World Health Organization, the proportion of the world’s population over 60 will almost double from 12% to 22% by the year 2050.^1^ Frailty is one of the greatest challenges facing an ageing population. Despite a significant number of studies in this area, there is no universally accepted definition of frailty. There is a consensus that frailty is a medical syndrome with multiple causes and contributors, characterized by reduced gait speed, weakness, low levels of activity, and exhaustion.^2^ Probably the most frequently used definition of frailty is the physical frailty phenotype proposed by Fried and colleagues,^3^ who characterized frailty in relation to 5 components; unintentional weight loss, low grip strength, self-reported exhaustion, slow walking speed and low physical activity. The presence of 3 to 5 of these characteristics identifies someone as frail.

The consequence of frailty is an increased risk of falls, delirium, disability, and death.^3-6^ Frailty is also a significant predictor of hospitalization and nursing home placement among community-dwelling older adults.^7,8^ While frailty can have devastating consequences, it is however dynamic, and can be reversible, with transitions occurring in either direction between non-frail, pre-frail and frail.

Frailty can be measured using a combination of physical, cognitive, psychological, social, and nutritional assessments. Fried’s Frailty Phenotype is a physical performance-based model that uses a combination of self-report and objective measurement and is validated for clinic and population screening with the ability to predict adverse outcomes.^9^ The Deficit Accumulation Index (DAI),^5^ or Frailty Index as it is commonly referred to, is based on the concept that frailty arises from the accumulation of health deficits. It incorporates a broad range of factors, across various domains such as physical function, cognition, self-rated health and biomarkers.^10^ Other frailty screening tools include the Edmonton Frail Scale^11^ which is a short and easy-to-administer tool assessing nine domains, including cognition, functional independence and polypharmacy.

Impaired physical function reflects reduced strength, mobility and balance which are core characteristics of frailty.^3^ Measures of functional performance such as the Berg Balance Scale,^12^ the Elderly Mobility Scale^13^ and the Timed Up and Go test^14^ are often used alongside other domains to gain a comprehensive understanding of an individual’s frailty status. However, the opportunities to undertake these assessments are limited to clinical visits, and there is the risk that the onset of frailty might go undetected or be identified too late. This delayed identification can result in missed opportunities for early intervention, thereby compromising the chances of maintaining functional independence.

Declining physical activity is also widely recognized as a key indicator of frailty, reflecting a gradual reduction in functional capacity and resilience in older adults.^15^ As people age, the loss of muscle strength and endurance – often referred to as sarcopenia – leads to diminished mobility and a reduced ability to perform daily tasks, which are hallmark signs of frailty.^3^ Studies show that decreased physical activity is associated with increased vulnerability to adverse health outcomes, including falls, hospitalization, and loss of independence.^16^ In fact, lower levels of physical activity not only predict the onset of frailty but also accelerate its progression.^9^ This reduction in movement often leads to a cycle of worsening physical and cognitive health, reinforcing the importance of early detection and intervention to preserve mobility and quality of life. Identifying and addressing this decline early can play a crucial role in preventing the more severe consequences of frailty and improving long-term health outcomes for older adults.^17^

Wearable sensors have become a pervasive means of measuring physical function and physical activity. A recent systematic review has shown that wearable sensors can be used to collect objective, quantifiable parameters of physical activity and physical function that can be used to distinguish between levels of frailty.^18^ This review found that parameters of physical activity and physical function obtained from a wearable sensor such as; time spent in non-sedentary activity, gait speed and balance correlate well with the identification of frailty. Most of the studies included in this review were conducted in laboratory or in experimental settings. However, considering that the signs and symptoms of frailty can be more subtle, regular or continuous evaluations in a person’s naturalistic, home environment may be more useful in identifying frailty. Giving older adults the tools they need to independently monitor for frailty may enable earlier intervention and therefore lower the likelihood of developing frailty. Early detection enables timely interventions, such as tailored exercise programmes, nutritional support, or medical treatment, which can slow down or reverse the progression of frailty.^16^ In addition, facilitating older adults to undertake these assessments independently in their own homes may also reduce the burden of testing, offering greater convenience for patients and minimizing the impact of health care on the daily routines. Furthermore, promoting self-monitoring fosters greater awareness and engagement in one’s own health, encouraging proactive management of age-related changes. Using wearable sensors to undertake these assessments may provide richer datasets, than the snapshot assessment obtained during visits to a health care facility. Such tools can bridge the gap between clinical visits, making frailty monitoring more continuous and accessible, especially for those with limited access to regular healthcare.

A major development is the use of machine learning algorithms to analyse data from wearables, enabling automated frailty detection. These models can process sensor data to identify physical activity and function indicators that distinguish frail or prefrail from robust with sufficient sensitivity and specificity.^19^ Despite these advances, challenges remain, particularly concerning data accuracy and questions remain as to whether consumer-grade wearables are precise enough to capture subtle declines in physical activity and physical function. Additionally, there is a need to explore whether assessments of physical activity and physical function can be performed in a home setting, providing a more realistic insight into how an older adult moves and whether they can use these devices in their daily lives.

We have previously reported on the ability of older adults to independently perform a TUG test in their own home, and capture objective test data using a research grade wearable sensor system.^20^ This study showed that the majority of individuals could successfully complete the TUG assessments unsupervised and obtain a frailty risk score using the wearable sensor system. This current study examined whether the sensor data captured independently by older adults can discriminate between frailty levels. Research-grade devices can provide granular data of physical activity and physical function. In this study we analysed data captured from both a researcher-grade wearable sensor system and a commercially available smartwatch to investigate if a consumer-grade device could provide data with sufficient detail to discriminate between stages of frailty.

## Methodology

A cross-sectional feasibility study was conducted with community-dwelling older adults which required participants to independently capture quantitative wearable sensor data during a physical function test and during free-living activities.

### Ethical considerations

The study protocol received ethical approval from the School of Health and Science Ethics Committee in Dundalk Insititute of Technology. Before beginning, each participant signed a written informed consent form.

### Participants

Participants for this study were recruited through advertisements in local golf, bridge and church community groups. Eligibility was assessed by a member of the research team over the telephone using the following criteria; 65 years of age and over, independently mobile, able to perform a series of mobility tests, had no cognitive or neurological deficits and no history in the past 6 months of lower limb orthopaedic trauma or surgery that would interfere with the ability to exercise. A sample of 52 was calculated based on power 0.8, effect size 0.8, and a *P*-value .05.^6^ The literature supports this with earlier research including comparable sample sizes.^19^

### Data collection

Participants were visited in their own home and a frailty assessment was conducted based on Fried’s Frailty Phenotype (FFP).^3^ The FFP classified participants as frail, pre-frail or non-frail. Participants then performed a Timed Up and Go (TUG) test^14^ under the supervision of the researcher. The TUG test evaluates the amount of time it takes a person to stand up from a typical chair, walk 3 m, turn 180°, walk back to the chair and sit down in seconds(s). It is a valid and reliable test of function and mobility.^14^

The Kinesis QTUG (Kinesis Health Technologies, Dublin Ireland) was used to provide objective test data during the TUG test. The QTUG provides an objective estimate of frailty in the form of a frailty risk score, which can be understood and used by the user without further analysis or interpretation required. The QTUG system relies on small, lightweight wearable sensors placed on the legs, which makes it unobtrusive and easy to wear, and does not require any complex setup or specialized knowledge, making it user-friendly even for older adults with limited technical skills.

Participants were instrumented with the QTUG sensors (Shimmer 9DoF Inertial Measurement Units, Shimmer Research, Dublin, Ireland) prior to performing the TUG test. The inertial sensors were secured around each shin, above the ankle joint using elastic tubular bandage and tape. Each sensor contains a tri-axial accelerometer, tri-axial gyroscope and a magnetometer. Data from each sensor were synchronized and processed using the Kinesis QTUG software application running on an Android tablet. Participants were requested to repeat the TUG test independently on the subsequent 2 days and obtain the objective frailty risk score using the QTUG system. Participants were instructed to follow the same 3 months course for the TUG test and to use the same chair as in the supervised test. Participants received training and were provided with a reference manual on how to undertake the TUG test independently and how to operate and use the QTUG system.

In addition to performing the TUG test unsupervised, participants were requested to wear the Withings ScanWatch (Withings, Issy-les-Moulineaux, France) for the subsequent 2 days to capture data during free-living activities. The Withings ScanWatch contains a tri-axial accelerometer for monitoring activity, as well as an embedded photoplethysmogram sensor for measuring heart rate and oxygen saturation, and electrodes for electrogram recording. In this study, only the accelerometer data were extracted from the ScanWatch for analysis. Participants were instructed to wear the ScanWatch around their non-dominant wrist, which was the hand less preferred for writing. Wearing a physical activity monitor on the non-dominant wrist is generally preferred because the dominant hand typically has more movement from tasks like writing, eating, or gesturing. This additional movement could lead to overestimation of physical activity.^21^ The ScanWatch was paired with a smartphone, however, this was not provided to participants during the 48-hour period.

### Data processing

Following the 48-hour period, the researcher collected all equipment from participants. The ScanWatch was synchronized with the Withings HealthMate app and data were downloaded for analysis. Twelve-hours wear time, during waking hours is considered sufficient to capture all relevant periods of activity, particularly in older adults.^22^ Therefore, to standardize the ScanWatch data, only data collected between the hours of 8am and 8pm were used and the following data on physical activity were extracted for analysis; total step count, maximum number of bouts and maximum sedentary time. The total step-count is the total number of steps detected during the selected timeframe. Step counts were selected as an intuitive metric of physical activity. Tudor-Locke and Bassett proposed a classification scheme for categorizing adult activity levels based on daily steps, describing individuals with less than 5000 steps per day as sedentary; 5000 to 7499 as low active; 7500 to 9999 as somewhat active; 10 000 to 12 499 as active; and >12 500 as highly active.^23^ Walking fewer than 3000 steps per day is associated with functional decline and frailty in community-dwelling older adults.^24,25^ A bout of activity is each unit of activity in 60-second epochs as identified by the watch when steps are detected. Maximum number of bouts refers to the number of consecutive activity detections in 60-second epochs and reflects continuous activity during a break in sedentary time. Sedentary time is defined as the duration between each bout of activity and is recorded in hours, minutes, and seconds. The maximum sedentary time is calculated for each participant and is the longest uninterrupted sedentary time that is, time between activity bouts when no steps are detected. Sedentary behaviour is associated with frailty and adverse health outcomes in older adults.^26^ Prolonged periods of inactivity, such as sitting or lying down while awake, with minimal energy expenditure can accelerate muscle weakness, joint stiffness, and poor mobility, which are central to frailty development.^27,28^

The Kinesis QTUG system collects raw data from the 2 wearable sensors during the TUG test. This raw data then undergoes multiple preprocessing steps to clean the signals and extract meaningful mobility metrics such as gait speed, stride length, postural sway, and phase durations. These metrics, along with the test subject’s demographic data, are then used by the QTUG software to produce a frailty risk score. Frailty risk scores produced by the QTUG below 50% are considered non-frail, values between 50 and 70% are considered transitionary, values above 70% are frail, while values above 90% are considered very frail. Validity of the underlying algorithms of the QTUG has been widely established.^29,30^ The frailty risk score obtained from the QTUG from the 2 unsupervised TUG tests were used in the analysis here.

## Data Analysis

Data analysis was performed using WEKA V3.8.6 (University of Waikoto, New Zealand). Machine learning classifiers were used to determine the influence of each variable in predicting frailty, focussing on establishing optimal thresholds for the QTUG frailty risk estimate and the 3 parameters extracted from the ScanWatch data (ie, total step count, maximum number of bouts and maximum sedentary time). The process began by using the mean of each variable as an initial reference for threshold selection, with the goal of identifying levels below which frailty risk, classified by the FFP, is predicted. The optimum threshold for each variable was defined as the point where the highest accuracy was achieved.

Following threshold selection, random forest models were applied to all variables. To evaluate the importance of each feature, an information gain attribute evaluation ranker was used. Three random forest models, representing different configurations with varying data subsets and parameters were implemented. The models’ robustness were tested using 10-fold cross-validation, which splits the data into 10 subsets. The models were trained on 9 subsets and tested on the remaining 1, repeating this process 10 times to ensure the models’ accuracy and reliability. This approach allowed for a thorough investigation of the predictive value of each variable for frailty.^31^

## Results

Fifty-one participants were enrolled in this study and the demographic characteristics of this cohort are presented in Table 1. Fifteen participants were unsuccessful in operating the QTUG system independently. Additionally, 4 participants declined to undertake the unsupervised component of testing. Therefore unsupervised frailty risk scores from the QTUG system were only available for n = 32 participants. ScanWatch data are presented for 45 participants. Data were missing for n = 6 participants due to: lost data by researcher error (n = 2), incomplete data obtained from the manufacturer’s web application (n = 2), and ScanWatch not provided to participants (n = 2) due to the presence of a permanent pacemaker, a contraindication for the smartwatch selected. All remaining participants had 12 hours wear time between the hours of 8am and 8pm for each day and were thus included in the analysis.

**Table 1.** Demographic characteristics of participants (n = 51).

Age (years)	Height (cm)	Weight (kg)	Sex	Frailty status
				Based on Fried’s frailty phenotype (FFP)
77.5 ± 8.4	163.6 ± 8.4	72.0 ± 13.5	Female 76%, n = 39	Frail 12%, n = 6;
				Pre-frail 61%, n = 31;
				Non-frail 27%, n = 14

Predictive accuracy, the number of correctly classified instances (CCI), sensitivity and specificity for each optimal threshold for each variable are presented in Table 2. Sensitivity is the ability of a test to correctly classify the presence of an outcome while specificity is the ability of a test to correctly classify the absence of an outcome.^32^ When examined individually, the unsupervised QTUG performed well in the prediction of frailty, with a threshold frailty estimate of between 35 and 40% providing a predictive accuracy of 75.8%, (CCI 22/29) (sensitivity 54.5%; specificity 72.2%).

**Table 2.** Predictive accuracy percentage and the number of correctly classified instances for each optimum threshold.

Variable	Threshold	% Accuracy	% Sensitivity	% Specificity	CCI n/29
Unsupervised QTUG frailty risk estimate	40-49	75	54.5	72.2	22
Step-Count	1300-1400	58.6	0	94.4	17
n-Bouts	150-200	51.7	18.2	27.8	15
MAX_ST (minutes)	55-58	68.9	27.3	83.3	20
Mean_ST (minutes)	8	72.4	9.1	100	21

Abbreviations: CCI n /29, correctly classified instances per total sample; n_Bouts, number of bouts of activity; MAX_ST, maximum sedentary time in minutes.

Confusion matrices and detailed accuracy for models incorporating data obtained from the unsupervised QTUG tests and the ScanWatch are presented in Table 3, while Confusion matrices and detailed accuracy for models incorporating physical activity data obtained from the ScanWatch only are presented in Table 4. The *y*-axis of the confusion matrix shows the actual classification of non-frail, pre-frail and frail. The *x*-axis indicates how participants were classified by the model. The detailed accuracy report gives an indication of the performance of the model or classifier. Precision, recall, *F*-Measure and ROC Area (receiver operating characteristic) are considered the most appropriate measures to include. Precision indicates the true positives that is, of all the data classified into each frailty cohort, what percentage of data actually belong there. Recall indicates the percentage of positives that were captured in each cohort. The *F*-Measure provides a weighted average of precision and recall while the ROC Area indicates the percentage of time the model would correctly classify a variable/participant. A measure of 0.8 is considered a strong result.^33^

**Table 3.** Prediction models and confusion matrices and detailed accuracy using ScanWatch and QTUG data.

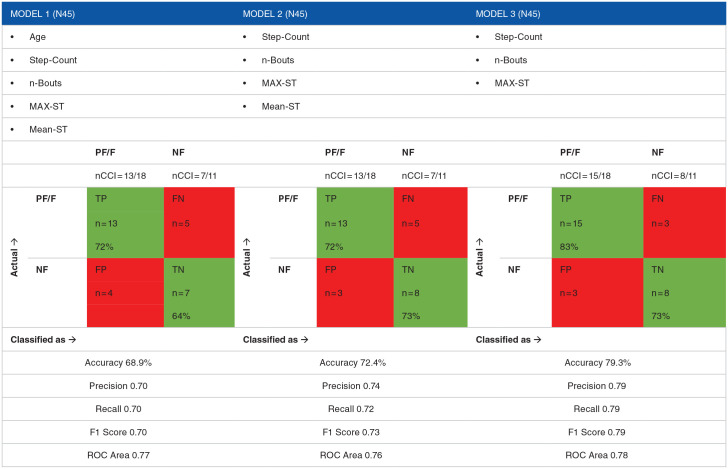

**Table 4.** Prediction models and confusion matrices and detailed accuracy using ScanWatch data only.

**Model 1 (n45)**					**Model 2 (n45)**					**Model 3 (n45)**				
• Age					• Step-Count					• Step-Count				
• Step-Count					• n-Bouts					• n-Bouts				
• n-Bouts					• MAX-ST					• MAX-ST				
• MAX-ST					• Mean-ST									
• Mean-ST														
CCI n33					CCI n33					CCI n33				
Confusion Matrix					Confusion Matrix					Confusion Matrix				
	**PF**	**NF**	**F**	**n CCI**		**PF**	**NF**	**F**	**n CCI**		**PF**	**NF**	**F**	**n CCI**
Actual →	23	3	2	**PF**	Actual →	23	4	1	**PF**	Actual →	23	3	2	**PF**
				n = 23/28					n = 23/28					n = 23/28
	5	7	0	**NF**		6	6	0	**NF**		5	7	0	**NF**
				n = 7/12					n = 6/12					n = 7/12
	2	0	3	**F**		2	1	2	**F**		2	0	3	**F**
				n = 3/5					n = 2/5					n = 3/5
**Classified as →**					**Classified as →**					**Classified as →**				
Accuracy 73.3%					Accuracy 68.9%					Accuracy 62.2%				
Precision 0.73					Precision 0.68					Precision 0.61				
Recall 0.73					Recall 0.69					Recall 0.62				
F1 Score 0.73					F1 Score 0.68					F1 Score 0.61				
ROC Area 0.79					ROC Area 0.80					ROC Area 0.77				

Because of the small sample size (n = 29) in the model incorporating the combined data, when classified into non-frail, pre-frail and frail there was only 1 participant in the frail group. For this reason, in the analysis of data from the QTUG and the ScanWatch combined, frailty status was distributed into a binary classification of non-frail and frail, with the frail group consisting of participants classified as pre-frail and frail. Prior to applying the machine learning analysis in the prediction of frailty, the information gains attribute evaluator ranked variables as follows; age, total step-count, number of bouts of activity (n-Bouts), maximum sedentary time (MAX_ST), mean sedentary time (Mean_ST) and unsupervised QTUG frailty estimate percentage. This ranking was used in the development of the prediction models presented.

## Discussion

This study examined quantitative physical function and physical activity data captured by older adults independently using wearable sensors and examined whether this data can be used to determine their frailty status. Physical activity data (total step count, maximum number of bouts and maximum sedentary time) were captured continuously for 48-hours during free-living activity using a commercially available smartwatch, the Withings ScanWatch, while physical function data were captured during a series of TUG tests using the Kinesis QTUG.

A machine learning classifier using 10-fold cross-validation was used to evaluate the performance of models derived from the parameters of physical activity obtained from the ScanWatch and from the Kinesis QTUG system and ScanWatch combined. All models incorporating the variables obtained from the unsupervised QTUG and the ScanWatch combined, performed better in the prediction of frail/pre-frail than non-frail as demonstrated in the confusion matrices, each with sensitivity and specificity ⩾ 0.70. This is an important finding as it has been shown that identifying people at the pre-frail stage as opposed to the frail stage increases the likelihood of a favourable transition between phases of frailty,^34,35^ therefore presenting an opportunity for appropriate distribution of limited resources for timely intervention. The QTUG data also performed well when analysed separately with the threshold of a frailty risk score between 35% and 40% providing a prediction accuracy of 75.8%. This is in keeping with the literature on the Kinesis QTUG system that supports its use in the classification of frailty.^36^ It is interesting to note however that despite the QTUG performing well in the prediction of frailty, when ranked using information gains evaluation for the development of prediction models, the QTUG variable was ranked last and the prediction model performed better when the QTUG data was omitted, with 79.3% accuracy, compared to 72.4%. One explanation for this may be the fact that the QTUG, while performed by the participants unsupervised in their own home, was performed in what could be perceived as test-like conditions and may have inadvertently resulted in response bias. The ScanWatch physical activity data was obtained in a continuous manner, without direct engagement required, which may have minimized any bias. As not all participants’ data were included in the analysis of the ScanWatch data and not all participants were able to successfully obtain a frailty score using the QTUG by themselves, the sample size (n = 29) used in the predictive models incorporating data from both the QTUG and the ScanWatch is reduced.

Models incorporating parameters of physical activity obtained from the ScanWatch performed best in the prediction of pre-frail with CCI n23/28 that is, accuracy of 82% in each model. This is consistent with the favourable prediction accuracy for pre-frail in the models incorporating the QTUG data. Thresholds derived from simple machine learning methods on each variable from the ScanWatch demonstrate predictive accuracy for frailty ranging between almost 52% for number of bouts of activity, 58% for total step-count and almost 70% for maximum sedentary time. A predictive accuracy percentage of 52% when analysed alone is of little value as chance is expected to achieve 50%.

The prevalence and technological advancement of wearables and smartphones have resulted in a notable surge in the accessibility of mobile applications and body-worn devices for health monitoring and evaluation in recent times. These technologies can be used to objectively and accurately assess the quantity and quality of movements in everyday and clinical settings, giving clinicians data that can be used to tailor, customize, and improve therapy. A more recent trend in the literature has been the increase in individuals self-monitoring information pertaining to their health and/or physical activity using wearables and illustrates the interest people have in monitoring and analysing their own data, referred to as quantified self-assessment.^37,38^ A self-directed, remote assessment of frailty status would enable prompt intervention and lower the likelihood of frailty, which would lessen the impact on both the individual and society as a whole. With its promise of genuine scalability, this technique has enormous potential to form part of community risk screening programmes that could use regular measurements taken by individuals in their homes.

This study has a number of limitations which should be considered. A sample recruited from community-based hospital clinics and social groups was expected to represent the target population of community-dwelling older adults. However few frail participants were recruited. This study took place during the COVID-19 pandemic which may have limited the recruitment of more frail older adults. Nonetheless, the sample’s combined 73% prevalence of frailty and pre-frailty is consistent with findings from other studies of a similar nature^39-41^ and reflects the population prevalence.^42^ The small sample (n = 51) included in this study is another limitation, and is unlikely to be powered sufficiently for machine learning analysis. Previous studies using machine learning have included sample sizes ranging from 240 to 5000,^43^ however other research conducted to examine the use of wearable technology to monitor gait and physical activity in frail older adults in the home environment have included similar numbers.^44^ While the results here should be regarded cautiously, this study does provide evidence to support the feasibility of deploying wearable sensors to older adults to capture physical function and physical activity data independently.

While wrist-worn devices offer a convenient and non-invasive way to monitor physical activity and movement, there are limitations to their use. Everyday hand movements, such as gesturing, typing, or eating, can be misclassified as physical activity, leading to overestimation of activity levels.^45^ To minimize this, participants were instructed to wear the ScanWatch device on their non-dominant wrist. While the QTUG system provides more detailed, quantitative data on mobility, the software interface is designed for clinician/researcher use, and is not intended to be administered by older adults. Many participants who attempted to use the QTUG system independently were unsuccessful for reasons including system or battery failure, poor eyesight and self-reported lack of confidence to attempt the test without support. The latter two reasons are related to biophysical restrictions and reduced confidence, both associated with ageing, and confirm the literature which identifies these as limiting factors to the use of technology among older adults.^46^ These challenges likely introduced bias by excluding or limiting the participation of individuals with more severe biophysical limitations or lower confidence levels. As a result, the findings may not fully represent the feasibility of the technology across the entire spectrum of older adults, particularly those living with frailty. Future studies should consider incorporating alternative assessment tools that are more user-friendly and specifically tailored to the needs of older adults to address these limitations.

Nonetheless, these findings are encouraging. They will guide future work to highlight to community-dwelling older adults the importance of early frailty recognition, emphasize the relevance of its identification to their independence and quality of life, and guide the development of a tool for older adults to use for remote continuous monitoring of their frailty risk.

## References

[1] Zhang Q Guo H Gu H Zhao X. Gender-associated factors for frailty and their impact on hospitalization and mortality among community-dwelling older adults: a cross-sectional population-based study. PeerJ. 2018;6:e4326. 10.7717/peerj.4326 PMC5834932 29507821

[2] Nahm FS. Receiver operating characteristic curve: overview and practical use for clinicians. Korean J Anesthesiol. 2022;75:25-36. 35124947 10.4097/kja.21209 PMC8831439

[3] Giggins OM Vavasour G Doyle J. Unsupervised Physical Function Testing Using a Wearable Sensor System – A Cross-sectional Study With Community Dwelling Older Adults. In: Salvi D Van Gorp P Shah SA eds. Pervasive Computing Technologies for Healthcare. PH 2023. Lecture Notes of the Institute for COmputer Sciences, Scoial Informatics and Telecommunications Engineering, Vol 572. Cham: Springer; 2024:438-448. 10.1007/978-3-031-59717-6_28

[bibr4-27536351241311845] Chang S-F Lin H-C Cheng C-L. The relationship of frailty and hospitalization among older people: evidence from a meta-analysis. J Nurs Scholarsh. 2018;50:383-391. 29874399 10.1111/jnu.12397

[bibr5-27536351241311845] Roe L Normand C Wren M-A Browne J O’Halloran AM. The impact of frailty on healthcare utilisation in Ireland: evidence from the Irish longitudinal study on ageing. BMC Geriatr. 2017;17:203. 28874140 10.1186/s12877-017-0579-0 PMC5583758

[6] AI-Therapy Statistics. 2018. https://www.ai-therapy.com/psychology-statistics/

[7] Kojima G. Frailty as a predictor of nursing home placement among community-dwelling older adults: a systematic review and meta-analysis. J Geriatr Phys Ther. 2018;41:42-48. 27341327 10.1519/JPT.0000000000000097

[8] Kojima G Taniguchi Y Iliffe S Jivraj S Walters K. Transitions between frailty states among community-dwelling older people: a systematic review and meta-analysis. Ageing Res Rev. 2019;50:81-88. 30659942 10.1016/j.arr.2019.01.010

[9] Dent E Kowal P Hoogendijk EO. Frailty measurement in research and clinical practice: a review. Eur J Intern Med. 2016;31:3-10. 27039014 10.1016/j.ejim.2016.03.007

[10] Morley JE Vellas B Abellan van Kan G , et al. Frailty consensus: a call to action. J Am Med Dir Assoc. 2013;14:392-397. 23764209 10.1016/j.jamda.2013.03.022 PMC4084863

[11] Sajeev S Champion S Maeder A Gordon S. Machine learning models for identifying pre-frailty in community dwelling older adults. BMC Geriatr. 2022;22:794-812. 36221059 10.1186/s12877-022-03475-9 PMC9554971

[12] Berg K. Measuring balance in the elderly: development and validation of an instrument. 1992. 1468055

[13] Tolley APL Ramsey KA Rojer AGM Reijnierse EM Maier AB . Objectively measured physical activity is associated with frailty in community-dwelling older adults: a systematic review. J Clin Epidemiol. 2021;137:218-230. 33915264 10.1016/j.jclinepi.2021.04.009

[14] Pradeep Kumar D Wendel C Mohler J Laksari K Toosizadeh N . Between-day repeatability of sensor-based in-home gait assessment among older adults: assessing the effect of frailty. Aging Clin Exp Res. 2021;33:1529-1537. 32930988 10.1007/s40520-020-01686-x

[15] Migueles JH Cadenas-Sanchez C Ekelund U. , et al Accelerometer data collection and processing criteria to assess physical activity and other outcomes: a systematic review and practical considerations. OA Sports Med. 2017;47:1821-1845. 10.1007/s40279-017-0716-0 PMC6231536 28303543

[16] Clegg A Young J Iliffe S Rikkert MO Rockwood K. Frailty in elderly people. Lancet. 2013;381:752-762. 23395245 10.1016/S0140-6736(12)62167-9 PMC4098658

[17] Podsiadlo D Richardson S. The timed “up & go”: a test of basic functional mobility for frail elderly persons. J Am Geriatr Soc. 1991;39:142-148. 1991946 10.1111/j.1532-5415.1991.tb01616.x

[18] Vijayan V Connolly JP Condell J McKelvey N Gardiner P. Review of wearable devices and data collection considerations for connected health. Sensors. 2021;21:5589. 34451032 10.3390/s21165589 PMC8402237

[19] Apsega A Petrauskas L Alekna V , et al. Wearable sensors technology as a tool for discriminating frailty levels during instrumented gait analysis. Appl Sci. 2020;10:8451.

[20] Gillespie LD Robertson MC Gillespie WJ , et al. Interventions for preventing falls in older people living in the community. Cochrane Database Syst Rev. 2012;2012:CD007146. 10.1002/14651858.CD007146.pub3 PMC8095069 22972103

[21] O’Halloran AM Hartley P Moloney D , et al. Informing patterns of health and social care utilisation in Irish older people according to the clinical frailty scale. HRB Open Res. 2021;4:54. 34240005 10.12688/hrbopenres.13301.1 PMC8220351

[22] Mitnitski AB Mogilner AJ Rockwood K. Accumulation of deficits as a proxy measure of aging. Sci World J. 2001;1:323-336. 10.1100/tsw.2001.58 PMC6084020 12806071

[23] Vavasour G Giggins OM Doyle J Kelly D. How wearable sensors have been utilised to evaluate frailty in older adults: a systematic review. J Neuroeng Rehabil. 2021;18:112. 34238323 10.1186/s12984-021-00909-0 PMC8268245

[24] Kojima G. Frailty as a predictor of hospitalisation among community-dwelling older people: a systematic review and meta-analysis. J Epidemiol Community Health. 2016;70:722-729. 26933121 10.1136/jech-2015-206978

[25] Tudor-Locke C Craig CL Aoyagi Y , et al. How many steps/day are enough? For older adults and special populations. Int J Behav Nutr Phys Act. 2011;8:80. 21798044 10.1186/1479-5868-8-80 PMC3169444

[26] Blodgett J Theou O Kirkland S Andreou P Rockwood K. The association between sedentary behaviour, moderate-vigorous physical activity and frailty in NHANES cohorts. Maturitas. 2015;80:187-191. 25542406 10.1016/j.maturitas.2014.11.010

[27] Greene BR Doheny EP O’Halloran A Anne Kenny R. Frailty status can be accurately assessed using inertial sensors and the TUG test. Age Ageing. 2014;43:406-411. 24212918 10.1093/ageing/aft176

[28] Parikh R Mathai A Parikh S Sekhar GC Thomas R. Understanding and using sensitivity, specificity and predictive values. Indian J Ophthalmol. 2008;56:341. 18158403 10.4103/0301-4738.37595 PMC2636062

[29] Hildebrand M VAN Hees VT Hansen BH Ekelund U. Age group comparability of raw accelerometer output from wrist- and hip-worn monitors. Med Sci Sports Exerc. 2014;46:1816-1824. 24887173 10.1249/MSS.0000000000000289

[30] Greene BR McManus K Redmond SJ Caulfield B Quinn CC. Digital assessment of falls risk, frailty, and mobility impairment using wearable sensors. Digit Med. 2019;2:125. 10.1038/s41746-019-0204-z PMC6906412 31840096

[31] Smith R. Validation and reliability of the elderly mobility scale. Physiotherapy. 1994;80:744-747.

[32] Pialoux T Goyard J Lesourd B. Screening tools for frailty in primary health care: a systematic review. Geriatr Gerontol Int. 2012;12:189-197. 22233158 10.1111/j.1447-0594.2011.00797.x

[33] Noah JA Spierer DK Gu J Bronner S. Comparison of steps and energy expenditure assessment in adults of fitbit tracker and ultra to the actical and indirect calorimetry. J Med Eng Technol. 2013;37:456-462. 24007317 10.3109/03091902.2013.831135

[34] McPhee JS French DP Jackson D , et al. Physical activity in older age: perspectives for healthy ageing and frailty. Biogerontology. 2016;17:567-580. 26936444 10.1007/s10522-016-9641-0 PMC4889622

[35] Pahor M Guralnik JM Ambrosius WT , et al. Effect of structured physical activity on prevention of Major Mobility disability in older adults: the LIFE study randomized clinical trial. JAMA. 2014;311:2387-2396. 24866862 10.1001/jama.2014.5616 PMC4266388

[36] Greene BR O’Donovan A Romero-Ortuno R , et al. Quantitative falls risk assessment using the timed up and go test. IEEE Trans Biomed Eng. 2010;57:2918-2926. 20923729 10.1109/TBME.2010.2083659

[37] Rockwood K Song X MacKnight C , et al. A global clinical measure of fitness and frailty in elderly people. Can Med Assoc J. 2005;173:489-495. 16129869 10.1503/cmaj.050051 PMC1188185

[38] Wang J Fu Y Lou V Tan SY Chui E. A systematic review of factors influencing attitudes towards and intention to use the long-distance caregiving technologies for older adults. Int J Med Inform. 2021;153:104536. 34325206 10.1016/j.ijmedinf.2021.104536

[39] O’Halloran A O’Shea M. Wellbeing and health in Ireland’s over 50s 2009-2016. 2018. 10.38018/TildaRe.2018-00

[bibr40-27536351241311845] Rawassizadeh R Momeni E Dobbins C Mirza-Babaei P Rahnamoun R. Lesson learned from collecting quantified self information via mobile and wearable devices. J Sens Actuator Netw. 2015;4:315-335.

[41] Rolfson DB Majumdar SR Tsuyuki RT Tahir A Rockwood K. Validity and reliability of the Edmonton frail scale. Age Ageing. 2006;35:526-529. 16757522 10.1093/ageing/afl041 PMC5955195

[42] Choi J Ahn A Kim S Won CW. Global prevalence of physical frailty by fried’s criteria in community-dwelling elderly with national population-based surveys. J Am Med Dir Assoc. 2015;16:548-550. 25783624 10.1016/j.jamda.2015.02.004

[43] Balki I Amirabadi A Levman J , et al. Sample-size determination methodologies for machine learning in medical imaging research: a systematic review. J Can Assoc Radiol. 2019;70:344-353. 10.1016/j.carj.2019.06.002 31522841

[44] Camerlingo N Shaafi Kabiri N Psaltos DJ , et al. Monitoring gait and physical activity of elderly frail individuals in free-living environment: a feasibility study. Gerontology. 2024;70:439-454. 37984340 10.1159/000535283 PMC11014463

[45] Jefferis BJ Parsons TJ Sartini C , et al. Objectively measured physical activity, sedentary behaviour and all-cause mortality in older men: does volume of activity matter more than pattern of accumulation? Br J Sports Med. 2019;53:1013-1020. 29440040 10.1136/bjsports-2017-098733 PMC6691867

[46] World Health Organization. Ageing and health. 2022.

